# Comparison of efficacy of Tranexamic Acid Mesotherapy versus 0.9% normal Saline for Melasma; A split face study in a Tertiary Care Hospital of Karachi

**DOI:** 10.12669/pjms.36.5.2379

**Published:** 2020

**Authors:** Sana Kaleem, Rabia Ghafoor, Sidra Khan

**Affiliations:** 1 Dr. Sana Kaleem, MBBS, FCPS II Trainee., Department of Dermatology, Jinnah Postgraduate Medical Centre, Karachi, Pakistan; 2 Dr. Rabia Ghafoor, MBBS, FCPS. Assistant Professor, Department of Dermatology, Jinnah Postgraduate Medical Centre, Karachi, Pakistan; 3 Dr. Sidra Khan, MBBS, FCPS II Trainee., Department of Dermatology, Jinnah Postgraduate Medical Centre, Karachi, Pakistan

**Keywords:** Melasma, Tranexamic acid, Mesotherapy

## Abstract

**Objectives:**

To compare the efficacy of tranexamic acid mesotherapy versus 0.9% normal saline for melasma by split-face study.

**Methods:**

It was a non-randomized clinical trial performed at the Dermatology ward of JPMC from September 2018 to June 2019 after getting approval from the Ethical Committee. A total of sixty patients were recruited in the study, who had symmetrical melasma on their faces. Both halves of the face were treated by Injection Tranexamic Acid (TA) with a dose of 4mg/ml and Normal Saline (NS) two weekly for twelve weeks. Hemi Modified Melasma Area and Severity Scoring (H-mMASI) was calculated at the start and end of the study. Analyses were done by SPSS version 23. P < 0.05 was taken as significant.

**Results:**

Mean of H-mMASI score was compared on both sides at the end of study, which showed significant reduction in mean score from 3.19 ±2.57 to 1.52 ± 1.2 (P < 0.05) on A side as compared to decline in scores on NS side from 3.46 ± 2.7 to 3.45 ± 2.6 (P > 0.05). Erythema, swelling, and burning were documented as temporary side effects on both sides.

**Conclusion:**

Tranexamic Acid (TA) mesotherapy can be considered as the most cost-effective, safe and directly observed therapy for melasma which showed significant improvement when old prior therapies have failed.

## INTRODUCTION

Melasma is an acquired disorder of hyperpigmentation of skin, which symmetrically affects the face.^1^ It is defined as dark hyperpigmented patches, which becomes more pronounced after sun exposure.^2^ Globally, prevalence of melasma ranges between 1–50%, while prevalence in Arab-American populations is 13.4–15.5%.^1^ No study has been conducted so far in Pakistan to determine the exact burden of melasma, however one study in Pakistan showed prevalence of 45% in pregnant women.^3^,^4^

Melasma is one of the chronic and relapsing disorders of skin. It causes depression and frustration in patients, which had worse impact on their psychosocial quality of life.^5^ There are different options available for the treatment of melasma which include topical agents, oral medicine, microinjections, mesotherapy and lasers. Recently, Tranexamic acid (TA) was introduced as a novel therapy for melasma.^2^

The rationale of using of intralesional TA for melasma is that it is cost effective, easy in use and free from systemic side effects in contrast to other traditional drugs used for melasma. Therefore, we conducted this study to see the efficacy and side effects of intralesional TA mesotherapy for moderate to severe melasma in comparison to Normal Saline (NS) as control in our population.

## METHODS

This non-randomized clinical trial was conducted from September 2018 to June 2019 in Department of Dermatolog, Jinnah Postgraduate Medical Centre Karachi, after getting approval from the Institutional Review Board (Ref.No: F.2-81/2018-GENL/2522/JPMC, Dated: 30-08-2018), sixty patients were enrolled in study. Sample size was calculated by NCSS software with 5% alpha error, 80% of power and 95% of the confidence interval.^6^ Patients were selected by purposive method of non-probability sampling technique. Written informed consent was taken from all patients. Those patients aged between 18 to 55 years with symmetrically moderate to severe melasma were included in study. Patients who had any medical illness, bleeding disorder, on anticoagulant therapy, pregnant females, lactating mothers or those who used topical treatment for melasma in last one month were excluded from the study.

Each patient was examined under Wood’s lamp and clinically diagnosed by consultant Dermatologists. All patients underwent pre-procedure assessment by complete history and physical examination. A comprehensive questionnaire was filled for each patient and color photographs were taken before sessions.

In this split-face study, we compared the efficacy of TA on left side with NS on right side of the face in the same patient. We calculated H-mMASI score for each half of the face before each session to determine the severity of melasma.^7^ H-mMASI is calculated by the percentage of involved area and darkness in patches of melasma.

Hemi-mMASI = forehead (0.15×D×A) + malar (0.30×D×A) + chin (0.05×D×A) A =Area =0-6

0=0%, 1=1%-9%, 2=10%-29%, 3=30%-49%, 4=50%-69%, 5=70%-89%, 6=90%-100%

D= Darkness of patches =0-4

0=absent or normal skin color without evidence of hyperpigmentation, 1=slight visible hyperpigmentation, 2=mild visible, 3=marked, 4=severe

Injection Transamine [Tranexamic acid=TA] of 500mg/5ml ampoule was used. With the help of insulin syringe of 100IU, 4IU (4mg) of TA and 96IU of NS were drawn which made the concentration of TA to 4 mg/100IU or 4 mg/ml. Initially face was cleaned by alcohol swab and then topical anesthesia (Eutectic mixture of local anesthetics, EMLA) was applied for 30-45 minutes. Multiple microinjections of TA were injected into patches of melasma 1 cm apart at left side of face by same insulin syringe.

At the same time, multiple microinjections of 0.9% NS by mesotheapry technique were injected into all patches of melasma on right side of the face. No other oral or topical cream was advised to use for home except sunblock.

At the end of this study means of H-mMasi score from right and left side of face were calculated separately and the mean of difference between both groups was compared. Side effects were documented at each follow-up. The response of patients towards improvement was graded by their self-level of satisfaction at the end of study as, Poor response rate= 0-25%, Fair response rate= 25-50%, Good response rate=50-75%, Excellent response rate=75-100%. We repeated the sessions every 2 weekly for 12 weeks. Patients were furthered followed up 12 weeks after end of study to document the maintenance effect of TA and recurrence of melasma.

### Statistical Analysis

Statistical Package for the Social Sciences SPSS Statistics ver. 23.0 was used for analysis of data. Categorical data were described in frequencies and percentages while numerical data were analyzed by means and standard deviation. The difference in means of H-mMasi score before and after the intervention for each side was analyzed by paired t-test. The difference in mean of H-mMasi score between the both sides after treatment was also analyzed by paired t-test. P<0.05 was taken as significant.

### Trial Registration Number

NCT04170088 by U.S. National Library of Medicine.

## RESULTS

On TA mesotherapy side, the mean H-mMasi score was 3.19 (±2.57) before treatment and 1.52 (±1.20) after 12 weeks of treatment, the difference in mean was 1.66 (± 2.22). Paired T test showed P-value of < 0.000 which shows significant improvement. On NS mesotherapy side, the mean H-mMasi score was 3.46 (±2.70) before treatment and 3.45 (±2.46) after 12 weeks of treatment, the difference in mean was 0.01 (± 0.15). Paired T test showed P-value of 0.483 which shows insignificant results.

**Fig.1:**
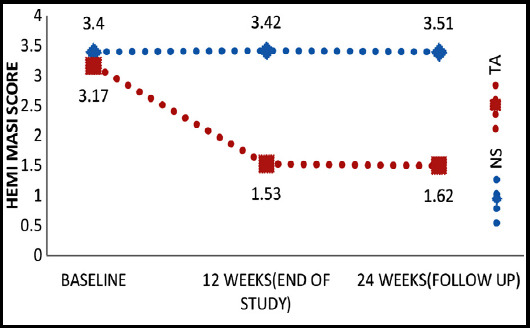
Comparison of Mean of Hemi-mMASI Score at TA and NS sides.

**Image-1:**
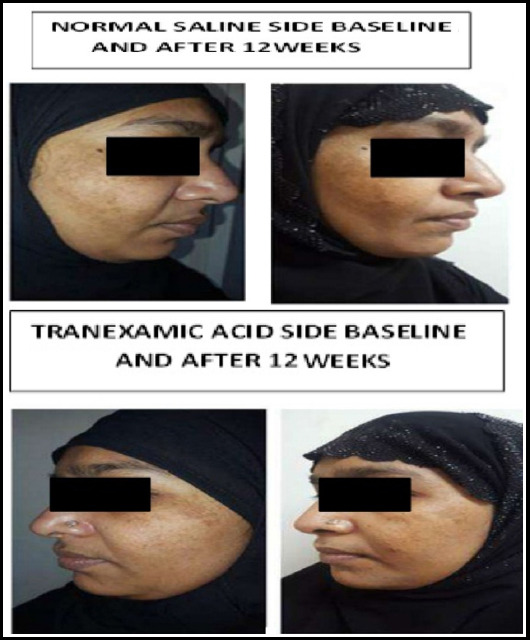
Comparison of Both Sides Before and After Treatment

Difference in means was calculated before and after treatment on both sides and then compared with each other by Paired t test which showed significant result by TA than NS, P-value <0.000. Hence, by both ways TA proved to be effective treatment for melasma as compared to NS. (Fig.1 & Image 1).

H-mMasi score remained 1.62 (±1.27) on TA side at follow up of patients after 12weeks of end of study which showed significant maintenance effect of TA as compared to NS. Hence TA proved to have low recurrence rate of melasma as NS with temporary side effects (Table-II).

**Table-I:** Demographic Data.

S.no	Demographic Data	Numbers	Percentages%
01	Mean Age	36±7.9	
02	Duration of Melasma	3.9 y ±2.02	
03	***Gender***		
	Female	54	90
	Male	6	10
04	***Marital Status***		
	Married	44	73.3
	Single	16	26.7
05	***Family History***		
	Positive	46	76.7
	Negative	14	23.3
06	***Fitzpatrick Skin Type***		
	Type IV	34	56.7
	Type III	24	40.0
	Type V	2	3.3
07	***Distribution of Melasma***		
	Centrofacial	40	66.6
	Malar	20	33.3
08	***Type of Melasma***		
	Dermal	33	27.5
	Epidermal	20	16.7
	Mixed	7	5.8
09	***Causes***		
	Pregnancy	13	43.0
	Cosmetic	9	30.0
	Oral contraceptive	6	20.0
	Hormone Replacement	2	6.7
10	***Previous treatment used***		
	Hydroquinone	36	60.0
	Skin whitening	20	50.0
	Laser	4	10.0

**Table-II:** Side effects of tranexamic acid mesotherapy.

Side effects	Numbers	Percentages%
Localized swelling	31	25.0
Erythema	6	5.0
Burning	8	6.1
Irritation	15	23.9

**Fig.2:**
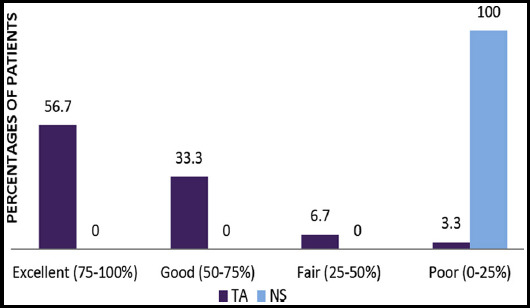
Comparison of Patient Satisfaction Level between TA and NS sides.

Out of 60, 4 patients showed excellent (60.7%), 34 patients showed good (56.7%), 20 patients showed fair (33.3%) and two patients showed poor (3.3%) response to TA, while all patients showed poor response on NS side. Hence, a total of 90% patients showed good to excellent satisfaction level at the end of study on TA side. (Fig-2)

## DISCUSSION

Melasma is one of prevailing pigmentery disorders of Asian skin. Its major clinical pattern is centrofacial which involves forehead, cheek, nose, chin, and lips.^1^ Different treatment modalities for melasma have been used in past including topical, oral medicine and interventional therapies.^8^ In 1979, Nijo reported a case of a patient of chronic urticaria treated with TA, showed improvement in melasma on face.^9^ After that many researches were done which showed the efficacy of TA for treatment of melasma in its oral, topical and intralesional forms.^10^ However, there are few studies of intradermal microinjection of TA for treatment of melasma in our population.^11^

On exposure of UV lights, keratinocytes induce melanogenesis and promote angiogenesis by activation of plasminogen activator (PA) thus increase skin pigmentation.^12^ TA causes inhibition of plasmin activation which leads to decreased activation of the melanocyte, reverses dermal vascularity and increases mast cell number hence reduce area, darkness, and homogeneity of patches of melisma.^13^,^14^

In France mesotherapy technique as ‘Localized Microinjection’ was first introduced by Pistor in 1979. It is defined as microinjections of 0.05 to 0.1 mL of highly diluted mixtures of drugs or a single drug in dermis or subcutaneous tissue of skin at the affected sites of the body which have medical or aesthetic issues. All intravenously injectable medicines can be used except for oily solvents and alcohol.^15^

To determine the efficacy of TA for melasma, many studies were done with or without other treatment in past. Lee et al.^11^ and Wu et al.^16^ described the efficacy of oral TA for melasma in their studies which showed 96% and 89% improvement in melasma respectively but side effects of oral TA like abdominal pain, headache and development of deep venous thrombosis were also noted. Kim et al signified the efficacy of topical 2% TA on melasma which showed improvement in 95% of cases with few side effects. But recurrence of melasma was determined few weeks later.^17^ Elfar et al. compared TA with glycolic peel in their study. Result was consistent with significant improvement in melasma in comparison to peel.^17^-^18^ In one study Saki et al compared the intradermal TA with topical 2% hydroquinone for melasma by split-face study, which showed significant improvement on both sides but no significant difference between both groups was observed. In contrast to our study, which determined the significant difference between TA and NS (P < 0.000).^19^ Our study documented the better efficacy of TA for melasma in contrast to NS and other studies, because TA reduced the pigmentation and surface area of patches (P< 0.000) significantly. It has temporary side effects with low recurrence rate of melasma.

Patients were further followed up to 12 weeks post-treatment to see long term effect of TA and recurrence of melasma. We observed that regimentation slowly appeared only in those patients who did not have strict sun protection. This finding was consistent with the study of Lee JH et al.^10^ A total of 90% patients showed good to excellent satisfaction level at the end of study on TA side same as Atefi et al. who also observed good satisfaction at the TA side as compared to hydroquinone.^20^

Common side effects of intradermal injection TA are visual abnormalities, hypotension, nausea, and vomiting reported in past.^21^ In our study no serious side effects were observed except temporary localized swelling, erythema, burning and irritation bilaterally, that was subsided after 24 hours with and without icing. Efar et al and Grimes also reported similar side effects of TA in their study.^18^,^22^ We observed that those patients who had positive family history, dermal type melasma and history of using hydroquinone were found resistant to TA completely. This could be secondary to hormonal or genetic factors and ochronosis.^23^ D’Elia et al and Aamir et al. also reported the same relationship of positive family history with resistant melasma.^24^,^25^

### Limitations of the study

It was single center study. In future further studies are recommended at multicenter level in our population.

## CONCLUSION

Intradermal microinjection of TA proved to be a better option for treatment of melasma, especially in resistant cases. It is one of the cost-effective therapeutic approaches which do not only increase compliance of patient towards treatment but also decrease the stress of patient by improving their quality of life.

### Authors’ Contribution

**SK:** Designing of study, data collection, study intervention, photograph taking, scoring, statistical analysis, writing, editing, approval of manuscript, critically revision.

**RG:** Supervised, helped in study design, selection of patient, statistical analysis, manuscript editing and final review of article.

**SiK:** Selection of patients, photographs taking, data collection, help in scoring and statically analysis, review of article.Go to:
